# Supplementary material for: Metabolomic and Proteomic Analyses to Reveal the Role of Plant-Derived Smoke Solution on Wheat under Salt Stress
Source: Int J Mol Sci. 2024 Jul 27;25(15):8216. doi: 10.3390/ijms25158216 (PMC11311447; doi:10.3390/ijms25158216)
Supplement: Supplementary file 1 [file ijms-25-08216-s001.zip › rev Figures S1-S5.pdf]

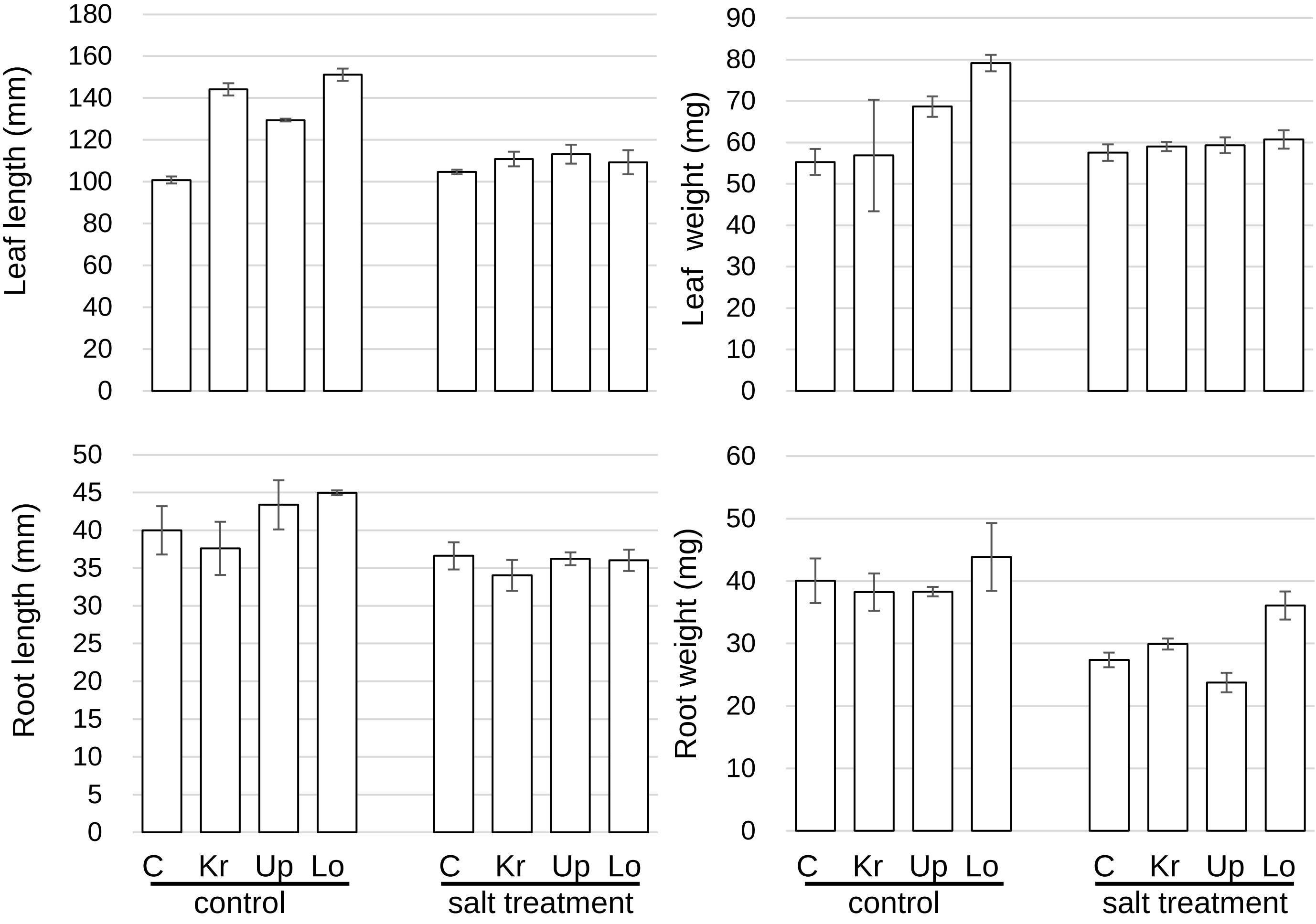

Figure S1. Morphological analysis of wheat treated with the upper layer and lower layer of plant-derived smoke solution under salt stress. Three-day-old wheats were treated with water (C), Karrikin(Kr), upper layer (Up), and lower layer (Lo) of plant-derived smoke solution with or without salt stress for 2 days. As morphological parameters, leaf length, leaf-fresh weight, main-root length, and total-root fresh weight were analyzed at 5 days after sowing. The data are presented as mean ± SD from 3 independent biological replicates.

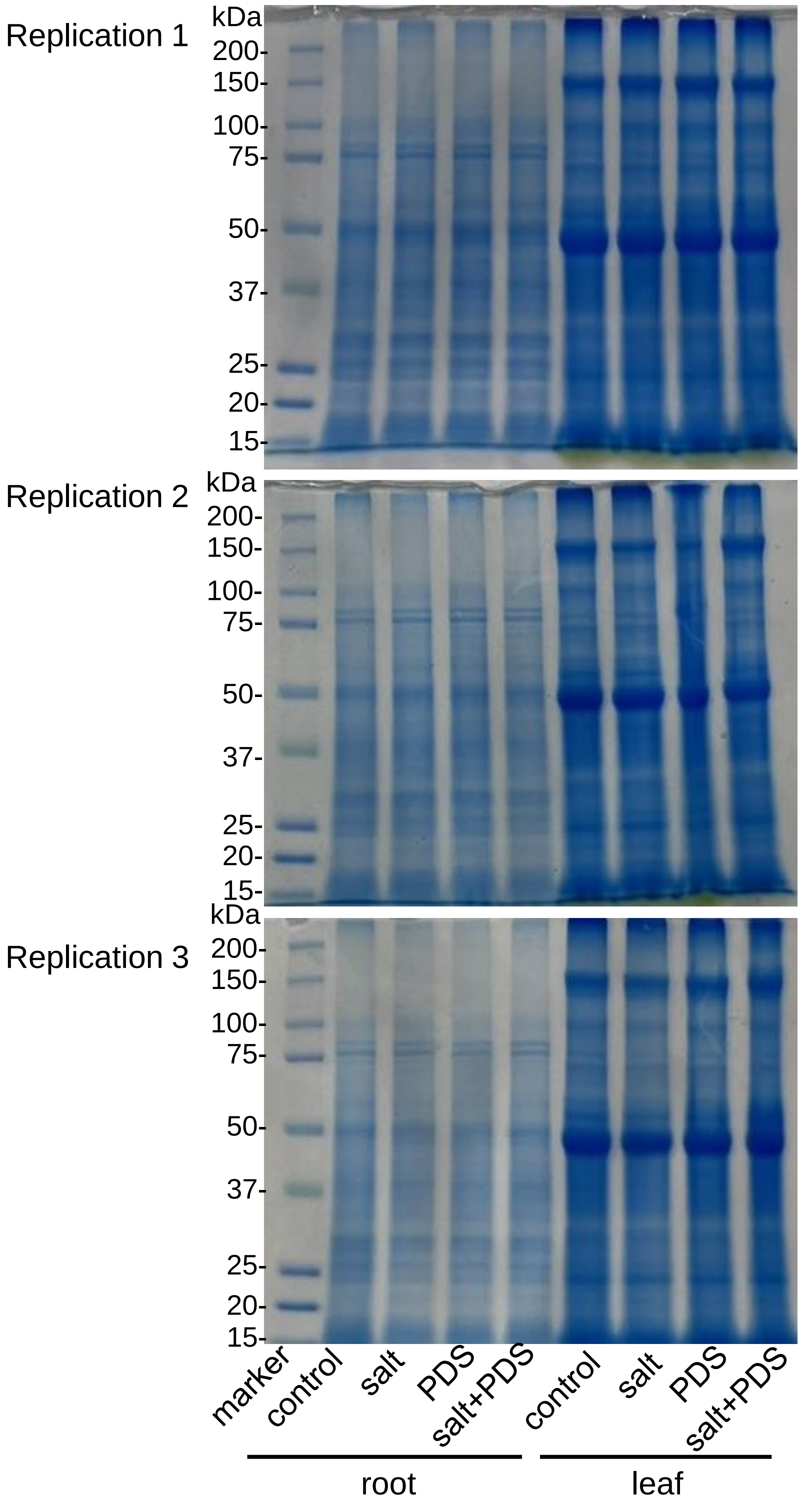

Figure S2. The Coomassie brilliant blue staining pattern of proteins used for immunoblot analysis. Experiments were performed with biologically triplicates for each treatments. Quantified proteins (10 µg) from roots and leaves were separated by electrophoresis on a 10% SDS-polyacrylamide. Coomassie-brilliant blue staining was used as a loading control.

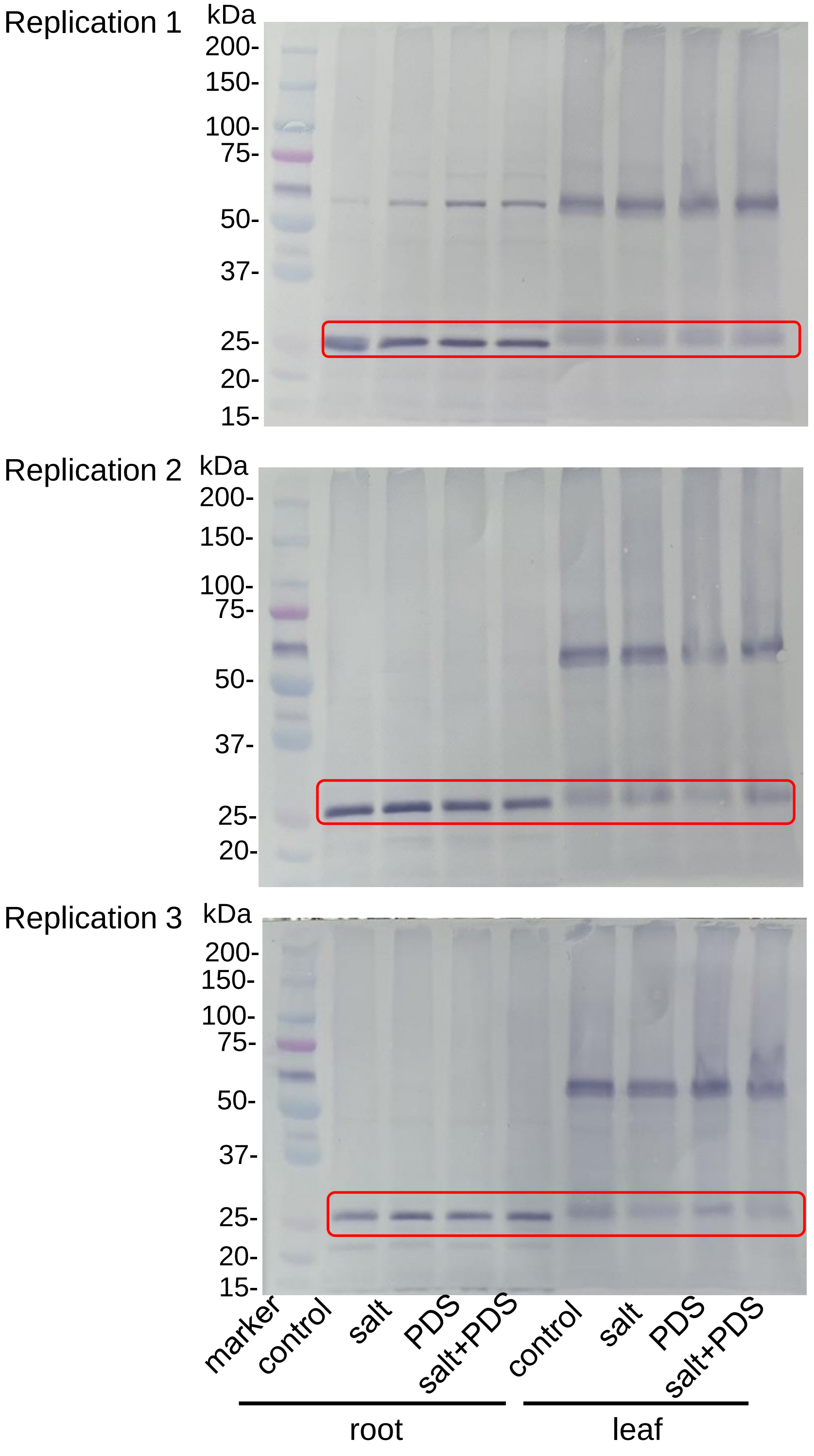

Figure S3. Blots of the entire membrane with anti-ascorbate peroxidase antibody, which were used in Figure 7A.

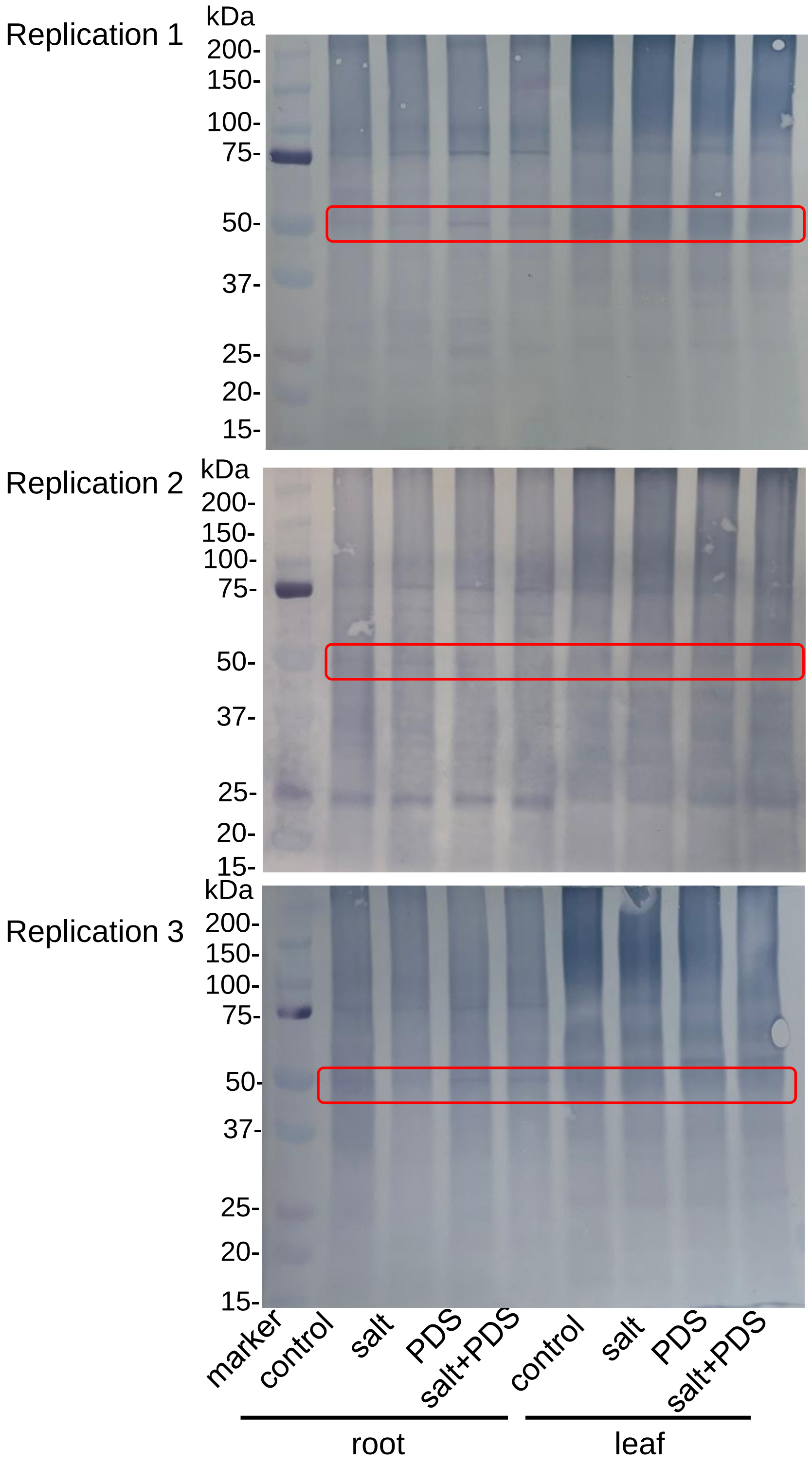

Figure S4. Blots of the entire membrane with anti-H^+-ATPase antibody, which were used in Figure 7B.

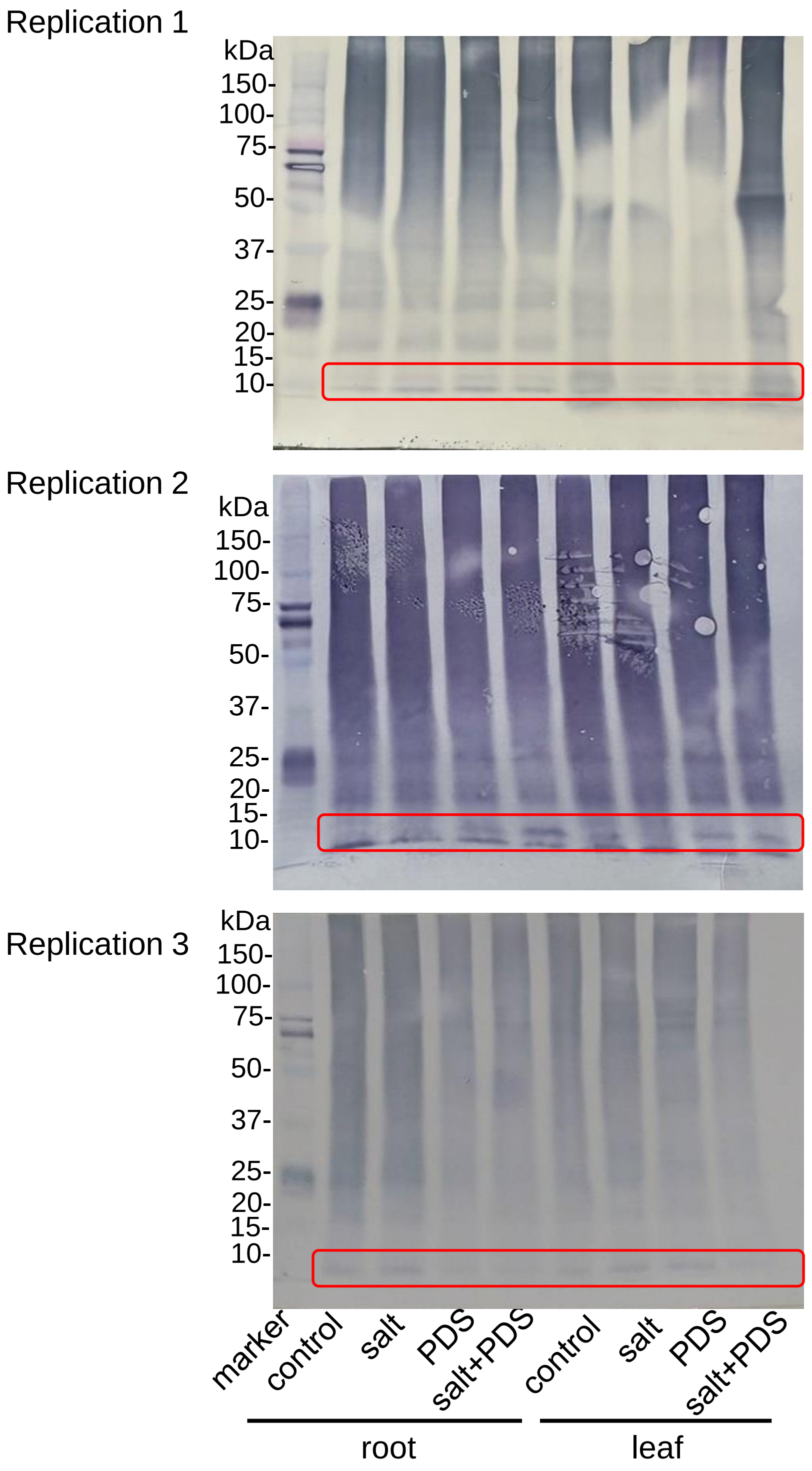

Figure S5. Blots of the entire membrane with anti-ubiquitin antibody, which were used in Figure 9A.
